# Perspectives on HIV Self-Testing Among Key and Affected Populations in Kenya

**DOI:** 10.4314/ahs.v22i2.5

**Published:** 2022-06

**Authors:** Neiloy R Sircar, Allan A Maleche

**Affiliations:** 1 Public Health Law Center; 2 Kenya Legal and Ethical Issues Network, Nairobi, Kenya

**Keywords:** Human Rights, HIV, Voluntary Counselling, Testing

## Abstract

**Background:**

Kenya's Key and Affected Populations (KAP) – men who have sex with men (MSM), female sex workers, people who inject drugs (PWID), and young women aged 18–24 – often experience stigma and discrimination in Kenyan health care settings due to their identity and/or behaviors, which can deter facility-based testing for HIV. Kenya has promoted self-testing as a means to reach these communities.

**Objectives:**

To identify KAP perspectives on self-testing and place our findings within Kenya's human rights and legal context.

**Methods:**

We conducted 4 focus group discussions (FGD) and 16 in-depth interviews (IDI). One FGD was conducted with each of the following communities: MSM, female sex workers, PWID, and young women aged 18–24. 1–4 IDI were conducted with each KAP community, and 1–3 IDI were conducted with health professionals working on HIV care in each study site. The semi-structured question guideline included one question soliciting opinions on self-testing.

**Results:**

KAP support self-testing in concept, however prevailing concerns among participants included access to pre- and post-test counseling services, as well as risk for harms (self-inflicted and otherwise) that might result from a positive result.

**Conclusion:**

Kenya should ensure that human rights are promoted and respected through implementing rights-based policies and practices for HIV self-testing, including pre- and post-test counseling.

## Introduction

Kenya has one of the highest burdens of people living with HIV (PLWH) worldwide, and for several years has been committed to identifying and treating PLWH.^1^,^2^ In service to this public health goal, Kenya's public health authorities adopted the principles of a human rights-based approach (HRBA) to HIV testing, notification services, and treatment.^2^–^4^ In addition, Kenya has passed a substantial human rights legal framework that can function to support HRBA if well implemented and enforced.^5^–^7^ Operationalizing HRBA to HIV programs, and developing indicators and tools to evaluate the implementation of HRBA, would provide a useful measure for how health and human rights can be better realized.^8^,^9^

A significant proportion of Kenya's HIV burden is carried by Key and Affected Populations (KAP): men who have sex with men (MSM), female sex workers, people who use injection drugs (PWID), and young women under 24 years of age.^1^,^10^–^12^ Kenyan public health authorities have invested significantly in outreach and capacity building efforts to connect KAP with testing and care services as part of their HIV and AIDS control program.^3^,^13^ However KAP are subject to significant burdens including stigmatization, discrimination, and under-realization of their human rights, despite Kenya's international obligations to promote human rights.^14^ Consequently, these populations might not trust or hold confidence in health providers to protect, respect, promote and fulfill their human rights to privacy, confidentiality, consent, and dignity when attending clinics and receiving health care, which could lead to under testing.^15^ Testing rates among some of these populations have been lower than targets: while 80–90% of female sex workers in Kenya report having tested for HIV within the past 12 months, only 77% of MSM, 84% of PWID, and about 50% of women aged 15–19 report having done so (though 80% of young women 20–24 reported testing).^1^,^3^,^10^,^13^

Understanding KAP perspectives and attitudes regarding HIV self-testing and incorporating that information into how Kenya's HIV programs and health care practices are developed and experienced, could help tailor improvements to these programs to better support HIV testing and promote KAP human rights. If these populations perceive that their rights are recognized then these rights may be enjoyed; whereas, if these populations perceive their rights are not recognized then a HRBA to HIV must incorporate overt and accountable human rights-enabling procedures as a matter of policy and practice.^16^,^17^

## Addressing stigma and discrimination through HIV self-testing kits

Self-testing kits have become a useful tool in Kenya and elsewhere in identifying new cases for HIV and facilitating connections to care and treatment.^14^ Global funders and HIV programs, including the U.S. President's Emergency Plan for AIDS Relief and UNAIDS, consider self-testing kits an important tool in accessing hard-to-reach populations and have encouraged their use across a variety of settings.^18^,^19^ In Kenya, self-testing kits have been deployed nationally to outreach those experiencing discrimination and stigmatization, and since 2017 these kits are available in public health facilities.^20^,^21^ Self-testing allows users to determine their HIV status within the privacy of their home or a similar context they may consider safe. Positive or negative results may be conveyed to a health care provider within a suitable facility for follow-up testing and linkages to counseling and if necessary treatment.^20^

Self-testing kits, in other countries with high HIV burdens, have led to increased testing rates and brought countries closer to Kenya and UNAIDS' “90-90-90” goals.^22^ Reaching, or exceeding, these goals will require increased testing amongst some communities at-risk for HIV, some of whom might be are criminalized in Kenya for their behaviors, and who suffer the consequences of religious, moral and cultural discrimination. Kenya's HIV and AIDS Prevention and Control Act (2006) does not envision self-testing kits, and as of this publication Kenya has not promulgated regulations to ensure the vested rights provided in that statute are incorporated into self-testing protocols.^6^ Kenya's 2017 self-testing operational guidelines note: “The potential for harm can be minimized if HIV self-testing is provided within a human rights framework, adequate information is provided, regulated and high- quality self- test kits are used, and there is adequate community involvement in decision making.” This is the only reference to a human rights framework within the document.^20^

## Methods

The protocol for the principal study is published.23 The issues surrounding self-testing were not the principal focus of the study, but enough discussions were generated to develop its own analysis. In summary this study was conducted in 2019–2020, and comprised four study sites in Kenya that corresponded to significant population centers for the four respective KAP communities: Nairobi (MSM), Mombasa (PWID), Kisumu (female sex workers), and Homa Bay (young women). These study sites also crrespond to counties with high HIV burdens.^12^,^13^

One focus group discussion (FGD), comprising 8–9 participants, and 1–4 in-depth interviews (IDI) were conducted with each population group. KAP participants were self-identifying members of those populations who were recruited with the assistance of community-based organizations that work with each respective community. Health care professionals working in Kenya's HIV care system, with PLWH and/or on HIV policy and programs, from each of the study sites were individually invited to participate in key informant interviews. We focused on this group of health care professionals as our goals was to assess HIV care within the context of self-test and human rights. Participants were compensated for local travel to the interview/discussion site. FGDs and IDIs were led by 1 interviewer per study site, each of whom were recommended by the community-based organizations as capable and trusted within the respective KAP. Fifty-two participants were included in the study. We refer to our published protocol for further detail as to our analysis and methodology,^23^ including our question guideline devised with support from KAP community-based organizations.

Data from FGD and IDI were recorded and transcribed. The discussions and interviews were conducted in English and Swahili, and led by a Kenyan interviewer who was a member of or otherwise trusted with the communities they engaged with. A Kenyan qualitative researcher with experience working on KAP- and HIV-related research provided secondary transcription, and conducted analysis of the qualitative data, producing analytic memos capturing themes and key results presented in each study site. Both authors reviewed all content and work products, and assessed the results from each study site independently (analytic memos), collectively (comparing study site results).

The main goal of the prinicpal study was to explore the implementation, or operationalization, of Kenya's HRBA to HIV testing and partner notification services.^24^ It posited that KAP trust and confidence in facility-based services (determined through KAP perspectives) could serve as an indicator for successful implementation as well as remaining challenges thereto. In that study, we suggested that KAP perspectives can act as an indicator for measuring KAP rights-realization (determined by the personal belief that one's rights are justly and consistently protected, respected, promoted, and fulfilled by public authorities). Our study was exploratory, and researchers adhered to a Grounded Theory approach for the qualitative analysis. We reviewed themes that emerged from our data and assessed those themes with respect to Kenya's laws and human rights obligations. The focus of this current paper is from one the themes that emerged during discussion surroundingthe question “How do you or how does your community feel about self-testing at home?” that was part of the interview and FGD guide.

Georgetown University's Institutional Review Board (2018-1148) and Kenya Medical Research Institute's Scientific and Ethics Review Unit (Non-KEMRI No. 654 (2019)) approved this study. NRS was an Afya Bora Consortium and NIH/Fogarty-Northern Pacific Global Health Fellow with the University of Washington and received funding through the University of Washington for this study.

## Results

Our results reflect an integration and synthesis of the information shared during the conduct of the principal study as it pertained to self-testing. We present the results by each KAP and within that the main themes. Individual quotes highlight key thoughts or contributions. We also include statements illustrating the occasions when alternative perspectives were shared.

Table 1 describes the participants per location. Thirty participants (57%) identified as female; 19 participants (36%) identified male; 3 participants were not identified by sex. MSM supported self-testing for reducing individual risk for stigmatization, but value counseling services connected with facility-based testing

**Table 1:** Participant in the qualitative data by study site and KAP community

Study site (KAP community)	Number of participants in focus group discussions (KAPs)	Number of participants in in-depth interviews (KAPs)	Number of participants in in-depth interviews (health care professionals)
*Nairobi (MSM)*	9	2	2
*Mombasa (PWID)*	9	3	2
*Kisumu (FSWs)* †	9	2	0
*Homa Bay (young* *women)*	8	2	4
**TOTAL** *	37	7	8

* n=52

† No suitable in-depth interviewees were identified or willing and able to participate in Kisumu. A small (n=2) group interview with FSWs independent from the focus group discussion provided similar outputs as the focus group discussion.

MSM were somewhat supportive of self-testing kits as an idea to learn one's status and link PLWH to care (Box 1). As homosexual behavior is illegal in Kenya, ensuring privacy and confidentiality was highly valued and particularly within the context of HIV testing. Repeated visits to clinics for testing raise concerns related to both learning one's HIV status as well as fear of discrimination for being a MSM. Especially in regions with few clinics, participants expressed worries that providers could become increasingly judgmental because of familiarity with a particular client. As one respondent stated, “OST [HIV oral self-testing] helps. Going for testing regularly is weird. It creates more stigma from clinicians.”

**Figure:**
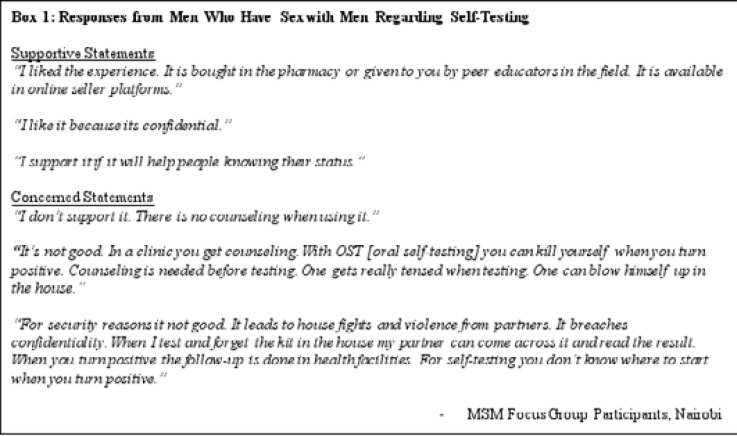


Counseling services were important to several MSM participants given risks for self-harm or violence from others should a self-test or its results be learned by partners and third parties. One MSM participant who engaged in sex work shared that sometimes “clients come with the self-testing kit and you are forced to test.”

Only health professionals interviewed in Nairobi mentioned self-testing in their interviews, within the context of strategies they considered important to ensure MSM (particularly) and KAP (generally) could test for HIV.

PWID were not supportive of self-testing and preferred facility-based services for their counseling services

PWID participants expressed their support for facility-based care (preferring community-based care providers over government-affiliated clinics) over self-testing broadly (Box 2). One FGD participant was particularly opposed to self-testing, with counseling as a major reason for why.

**Figure:**
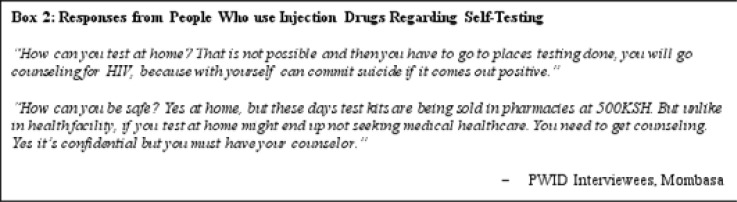


Interviewer: So, you think this self-testing can bring discrimination?

Group Participation: (in uniformity) Yes. This is not good.

Interviewer: How will it bring discrimination?

Respondent: No one has been told anything regarding HIV testing, because there are steps in sensitizing: you must be counseled first and prepared on the outcomes and how you shall be living, if found positive. But if you test with your partner alone, can start a fight and all reveal everything to the neighbours.

Access to counseling and services with respect to testing, learning of one's status, and if necessary going onto treatment, was important for these participants. Privacy, while also important, carried risks that a positive result from a self-testing kit could result in self-harm and suicide.

### Female sex workers value in-clinic counseling significantly

Female sex worker participants were generally supportive of self-testing kits in principle, believing that the convenience, privacy, and the ability for a user to test themselves as frequently as they preferred were advantageous. One participant said that self-testing could be popular with sex workers, as “they will like it because it is something in their possession, and you can test yourself in a room at any time without the knowledge of your colleagues. It can be good.” Another participant conveyed her trust in the quality and accuracy of self-testing kits, stating that, “the machine has already been tested and qualified that it works.”) Sex work being illegal in Kenya influenced preferences for minimal risks to being placed in jeopardy. Concerns for self-harm and suicide were also manifest in this group, but less prevailing as compared to other communities. A female sex worker participant had a similar comment to one of the MSM participants: “[A] man came, tested, was given a kit; he went the home and forced the girl – the wife – to test.” While clients and partners testing for HIV is a good health practice and respectful to those parties involved, being forced or coerced to do so is not.

Sex workers however expressed preference for counseling services as part of the HIV testing experience and were concerned that self-testing would not provide these resources readily (Box 3). Participants felt stigmatization was still an impediment to testing, especially with government facilities, and so where they would acquire self-testing kits was pertinent to their uptake among their community. Participants said distributing self-testing kits through trusted distribution methods – peer-to-peer and community-based organizations – was better than receiving the self-testing kits from government-affiliated clinics and hospitals.

**Figure:**
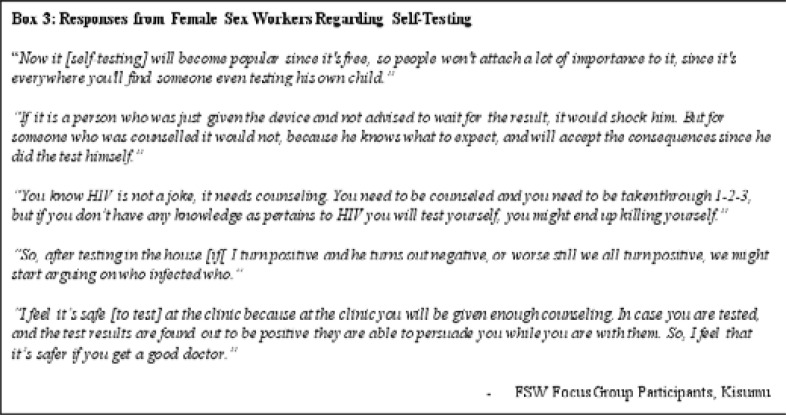


Young women supported self-testing as a means to ensure privacy, but prefer clinics for services and shared concerns over self-harm and suicide (Box 4)

**Figure:**
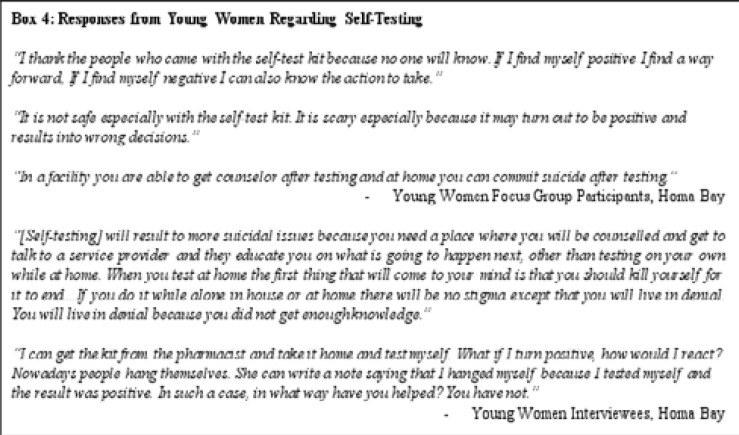


Some young women participants supported self-testing kits as they provided a way to avoid travel to and entering HIV testing centers. Participants noted that in rural communities in western Kenya association with an HIV testing center was sufficient for social discrimination (e.g. gossip) about a person. However, as with other KAP, young women participants were generally more supportive of facility-based care or other community-based care including a health care provider assisting with testing at home. Access to counseling and other care services were an important determination in support for using self-testing kits, particularly as a positive result might result in self-harm and suicide.

## Discussion

As with earlier studies,^25^–^27^ our findings suggest that self-testing kits might serve a useful role in increasing HIV testing rates among KAP and initiating patient-driven linkages to facility-based testing and treatment.^22^,^28^ Multiple studies, including Knight et al (2017), demonstrate that self-testing strategies in sub-Saharan Africa can be successful and well received by hard to reach populations. 29 At the same time, though, we found results that indicate hesitancy to use these products that other studies have not been previously highlighted or thoroughly explored.^28^, ^29^ Within the Kenyan context, self-testing kit uptake and the strategy for linking test-takers to care-givers could improve if the self-testing strategy includes universal access to counseling, which Kenya's law requires,^6^ while optimizing care.

Kenya, at the time of our study, did not have a national or coordinate mental health hotline; with the COVID-19 crisis, public health authorities have begun to build one.^30^ This apparatus could be accessible at all times to persons who need counseling, including persons who self-test for HIV, and be equipped with specialists for that purpose. Similarly, Kenya has a help line for persons who struggle with alcohol and drug abuse which provides a model and infrastructure that could lend itself to HIV related mental health needs as well.^31^

Facility-based care is preferred by several KAP participants over self-testing kits on account of access to pre- and post-counseling

KAP participants expressed concern about self-harm and suicide if a self-tester was HIV positive and no pre- or post-counseling services were in place; some participants noted that it could also result into family or partner abuse, with a high potential for harm (from others, or self-inflicted). Pre- and post-counseling services are an essential component of HIV testing, treatment, and care; importantly, such services are a guaranteed right under Kenyan law.^6^ Participants were unclear how such services are provided in conjunction with self-testing kits, suggesting the implementation of Kenya's self-testing strategy can improve through greater information dissemination.^20^ Noted above, an information hotline that preserves anonymity and confidentiality – and could also provide pre- and post-test counseling over the phone or online – might support Kenya's self-testing strategy.

Self-testing kits could become coercive tools in the absence of suitable support structures

Comments from MSM and female sex workers suggest that self-testing kits could be used coercively within the home, or elsewhere, to test partners and other persons - a situation that Kenya's public health authorities should consider as part of the self-test kit strategy since the program itself could potentially contribute to rights violations. Inadequate monitoring of the impact of the self-testing program may lead to missed opportunities to identify and or address instances of wrongful use, the reporting of which might also be included in a national hotline service.

Kenyan law forbids coercive HIV testing,6 and the 2017 self-testing guidelines recognize this risk,^20^ yet some of our participants suggest accountability mechanisms need strengthening. If the coerced person has few means or resources to press for their rights then the mere existence of those rights on paper is ineffectual; rights may only be realized from the perspective of those who hold them.

Self-testing kits may not reach certain populations if distributed in a manner that impedes their access and use

Several participants from the sex worker FGD felt that using peer-to-peer mechanisms (including through community-based organizations) to distribute self-testing kids would be preferable than relying on other distributors including pharmacies and government-affiliated clinics or hospitals. This reaffirms our other analyses of KAP trust, or lack thereof, for government-affiliated services and programs.^23^ While self-testing kits are able to reduce risks for stigma in attending a facility – as some young women and MSM noted – they do require individuals to acquire (and dispose of) them in manners that could breach privacy - such as in-person pharmacy procurement, mail delivery with recognizable packaging for kits, or disposal that could lead to a third party attaining protected information. Even where self-testing kits are effectively deployed to mitigate the aforementioned risks, linking a test-taker to hospitals and clinics might be impaired on account of a lack of social support, and KAP negative perceptions of government-affiliated health centers may impede their attending facilities.

### Self-testing kits must be linked to follow-on testing

Self-testing kits tend to be very accurate,^32^ and so a high degree of trust is warranted and makes such kits valuable in resource-constrained contexts. Nevertheless, follow-on testing with a health care provider would be essential to verify results. A false positive or false negative outcome would be unquestionably devastating. As indicated by several respondents in our study sites the results from a first self-test may be trusted in isolation, and while that could lead users to seek follow-on testing and treatment in an HIV clinic it may lead others elsewhere, including to harm.

## Limitations

Our data collection took place over in 2019 pre-COVID, and comprised a small sample of participants and was limited to those who were accessible in our study sites. One qualitative researcher and our interviewees transcribed collected data, and our qualitative researcher provided analysis in tandem with the two authors who are principally trained as lawyers. Our study's main goal and data collection did not focus on HIV self-testing kits or Kenya's self-testing strategy, and so our results are drawn from what participants said on their own or in response to minimal probing, i.e. one question, from interviewers and FGD. Differing views may have been held among participants but not detected. As with all qualitative studies, generatability of these findings to all KAP is not possible. Nonetheless, future assessment of the self-testing strategy in Kenya should take into account the views expressed by participants in this study to understand barriers and facilitators in the use of self-test to reach the 90-90-90 goals.

## Conclusion

Our study indicates that Kenyans' right to effective pre- and post-counseling programs must accompany Kenya's self-testing strategy, so that a test-taker is linked to suitable services before and after self-testing. Referencing a human rights framework or the existence of HIV testing guidelines is insufficient to ensure that said framework or guidelines are put into practice and experienced routinely. Equally important, Kenya should consider its legal obligation under the HIV and AIDS Prevention and Control Act and develop amendments to that statute to account for self-testing as well as regulations to ensure the law's enforcement in a manner that promotes safety, privacy, and confidentiality. It is insufficient to simply sever HIV testing from facilities and clinics and presume that at home testing will be adequately private and confidential, and further that self-testing will always and only occur under optimal conditions with respect to the test-taker's rights. The 2017 self-testing guidelines call upon programs to consider context-specific approaches – that contextual understanding should include why KAP are reluctant to test in the first place and what drives their reluctance, and with self-testing that contextual approach should recognize and reinforce Kenyan rights.
